# Detection of Carbapenem-Resistance Genes in *Klebsiella* Species Recovered from Selected Environmental Niches in the Eastern Cape Province, South Africa

**DOI:** 10.3390/antibiotics9070425

**Published:** 2020-07-21

**Authors:** Kingsley Ehi Ebomah, Anthony Ifeanyi Okoh

**Affiliations:** 1 SAMRC Microbial Water Quality Monitoring Centre, University of Fort Hare, Alice 5700, South Africa; AOkoh@ufh.ac.za; 2 Applied and Environmental Microbiology Research Group (AEMREG), Department of Biochemistry and Microbiology, University of Fort Hare, Alice 5700, South Africa

**Keywords:** carbapenem-resistance genes, *bla_KPC_*, *bla_OXA-48-like_*, *bla_NDM-1_*, *K. pneumoniae*

## Abstract

Carbapenemase-producing Enterobacteriaceae (CPE) have been heavily linked to hospital acquired infections (HAI) thereby leading to futility of antibiotics in treating infections and this have complicated public health problems. There is little knowledge about carbapenemase-producing *Klebsiella* spp. (CPK) in South Africa. This study aimed at determining the occurrence of CPK in different samples collected from selected environmental niches (hospitals, wastewater treatment plants, rivers, farms) in three district municipalities located in the Eastern Cape Province, South Africa. Molecular identification and characterization of the presumptive isolates were determined using polymerase chain reaction (PCR) and isolates that exhibited phenotypic carbapenem resistance were further screened for the possibility of harbouring antimicrobial resistance genes. One hundred (43%) of the 234 confirmed *Klebsiella* spp. isolates harboured carbapenem-resistance genes; 10 isolates harboured *bla_OXA-48-like_*; 17 harboured *bla_KPC_*; and 73 isolates harboured *bla_NDM-1_*. The emergence of *bla_KPC_*, *bla_OXA-48-like_*, and *bla_NDM-1_* carbapenem-resistance genes in *Klebsiella* species associated with environmental sources is of great concern to public health.

## 1. Introduction

Antimicrobials have been considered to be on the losing side in the therapeutic battle against pathogenic microorganisms, nevertheless, carbapenems (regarded as the drugs of last resort) are the preference for severe infections caused by antimicrobial resistant bacterial strains [1]. Carbapenem-resistance Enterobacteriaceae (CRE), including *Klebsiella pneumoniae*, generally possess mucoid which is one of the most important known nosogens that causes urinary tract infections, wound infections and septicaemia in immuno-compromised patients. Reports have shown that CRE infections are also associated with patients that have been exposed to high use of antimicrobial agents or have engaged in transplantation of organ/stem cell, mechanical ventilation, and possibly extended stay in hospitals [2]. CRE can cause treatment failure thereby constituting a significant public health concern [3,4].

The resistance against antibiotics represents an important problem because the phenomenon involves different bacterial species which include *Pseudomonas aeruginosa* [5] and *Escherichia coli* [6]. Antimicrobial resistance genes (ARGs) can be transferred to other bacterial species and thereafter can lead to the production of various enzymes (such as beta-lactamases) that inactivate antimicrobial activities; the types of enzymes are myriad in carbapenemase-producing carbapenem-resistance Enterobacteriaceae (CP-CRE), and include clavulanic-acid-inhibited β-lactamases (Class A: *Klebsiella pneumoniae* carbapenemase, KPC; non-metallocarbapenemase, NMC; imipenemase, IMI; *Serratia marcescens* enzyme, SME; and Guiana extended-spectrum, GES), metallo-β-lactamases (Class B: Verona integron-encoded metallo-β-lactamase, VIM; New Delhi metallo-β-lactamase-1, NDM-1; German imipenemase, GIM; and Sao Paulo metallo-β-lactamase-1, SPM-1) and extended-spectrum oxacillinases (Class D: oxacillinase OXA-48-like) [7,8,9]. However, members of Enterobacteriaceae group also produce class C enzymes (chromosomal or acquired) and extended spectrum beta-lactamases (ESBLs) that can confer antimicrobial resistance (AR) against carbapenems when combined with mutations in chromosomal porin that avert accumulation of β-lactam agents in the bacteria [10,11]. Additionally, microbial resistance against carbapenem has become a global concern, and studies on the detection of carbapenemase-producing *Klebsiella* spp. (CPK) isolates in clinical settings are increasingly being reported [12,13]. Several studies including Waseem et al. [14] have reported CPK and ARGs in non-clinical environments, Proia et al. [15] (2018); Khan et al. [16] in wastewater treatment plants (WWTPs), Uyaguari et al. [17], in agricultural samples and Stange et al. [18] in rivers and lakes. However, none of these studies investigated at the same time the occurrence of relevant carbapenem-resistance genes (CRGs) in isolates recovered from environmental samples. Hence, our current study was aimed at determining the occurrence of *Klebsiella* spp., the antimicrobial susceptibility patterns and the presence of three relevant CRGs such as *bla*_KPC_, *bla*_NDM-1_, and *bla*_OXA-48_.

## 2. Materials and Methods

### 2.1. Description of Study Areas and Sample Collection

**Figure 1:**
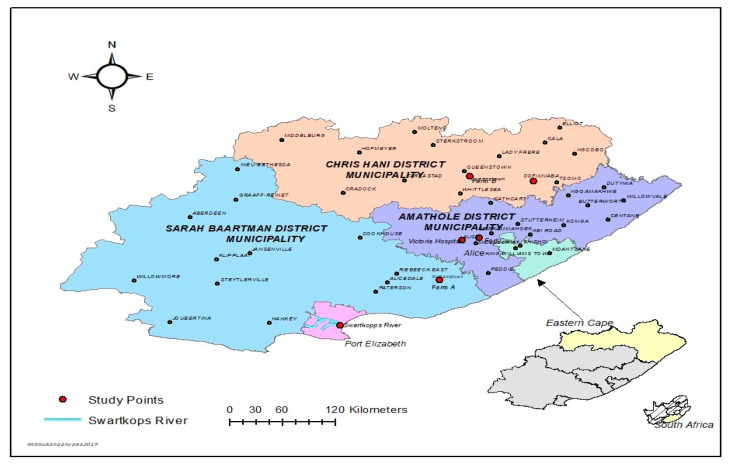
Map showing the study areas in the district municipalities.

The sampling sites comprise of hospitals, WWTPs, surface waters (rivers, dams, canals), and farms within villages and towns in Amathole District Municipality (DM), Sarah Baartman DM, and Chris Hani DM, in the Eastern Cape Province (ECP), South Africa. Four WWTPs, three hospitals and two farms were used as sampling sites for this study. Different samples (243 in total) comprising surface water (47), WWTPs final effluents (29), hospital effluents (27), irrigation water (45), soil (60), and vegetables (35) were aseptically collected once-off between the months of January 2018 and September 2018. Water samples were collected in duplicate using sterile 1-L glass bottles and transported in an ice box to the laboratory for microbiological analyses. Vegetable samples including spinach, broccoli, and cabbage were randomly collected from selected farms in the study areas. Figure 1 is a map showing the study areas within the DM in the ECP, South Africa.

### 2.2. Sample Preparation and Processing

#### 2.2.1. Processing of Vegetable Sample

For the processing of vegetable samples, the method of Du Plessis et al. [19] was adopted. Briefly, 10 g of each vegetable sample was placed in 90 mL of sterile Trypticasein Soy Broth (TSB) in a Stomacher Bag and was macerated with Mixer machine, and 90 mL of the broth was incubated at 37 °C for 18–24 h in sterilized bottles. Thereafter, a loopful of TSB was streaked on the surface of MacConkey agar plates, incubated for 24 h at 37 °C. From which, four single colonies from the overnight culture were streaked on nutrient agar (NA) plates, incubated for 24 h at 37 °C. Afterwards, glycerol stock was prepared from the NA plates in nutrient broth (NB).

#### 2.2.2. Processing of Soil Sample

The method of Da Silva et al. [20] was also adopted for biological analysis of the soil samples. Sixty agricultural soil samples were randomly collected from selected farms in the study areas. A total of 10 g of each soil sample was suspended in 90 mL of sterile TSB and incubated at 37 °C for 18–24 h. Thereafter, a loopful of TSB was streaked on MacConkey agar plates and were incubated for 24 h at 37 °C. Purification process was carried out on NA plates as described above.

#### 2.2.3. Processing of Water Samples

To isolate the enteric bacteria from the water samples, the membrane filter technique [21] was used. From each water sample, 100 mL of the water samples was filtered through a sterile membrane filter of 0.45-μm pore size. Thereafter, the membrane filters were aseptically picked, placed onto the surface of MacConkey agar plate, and incubated at 37 °C for 18–24 h. Four colonies were picked for isolation. After incubation, presumptive *Klebsiella* spp. isolates were streaked onto NA plates and incubated at 37 °C for 24 h. Thereafter, 25% glycerol stock was prepared from the cultured broths and preserved at −80 °C for further analyses.

### 2.3. DNA Extraction

Bacterial DNA was extracted by performing the boiling method as described by Jackson et al. [22]. Briefly, 4 mL of NB was prepared and presumptive isolates were resuscitated and also extracted from the broth cultures following the method as described in a previous study [23]. The DNA templates were stored at −20 °C for further molecular analyses.

### 2.4. Molecular Identification of Presumptive Isolates

The presumptive isolates were confirmed by polymerase chain reaction techniques (PCR) and PCR products were viewed using agarose gel electrophoresis (AGE). The list of primers used and reaction protocols are listed in Table 1.

### 2.5. Antimicrobial Susceptibility Test of the Confirmed Klebsiella Species

All confirmed *K. pneumonia* and *K. oxytoca* isolates were subjected to antimicrobial susceptibility test against a panel of four carbapenems following Kirby–Bauer disk diffusion method and results were interpreted according to the Clinical and Laboratory Standards Institute [22] guidelines. The list of carbapenem antibiotics includes doripenem (10 µg), imipenem (10 µg), meropenem (10 µg), and ertapenem (10 µg). Briefly, about 100–200 µL of the bacterial overnight broth was transferred into 5 mL normal saline solution which was adjusted matching 0.5 McFarland standard. Thereafter, 100 µL was spread on Muller–Hinton agar plates with the use of sterile glass spreader, and plates were impregnated with the aforementioned carbapenem antimicrobial discs, incubated aerobically at 37 °C for 24 h. After which the diameters of zone of inhibition were measured and interpreted according to the recommended criteria by the CLSI [25].

### 2.6. Molecular Characterization of the Relevant Carbapenem Resistance Genes

All strains of *Klebsiella* spp. that exhibited phenotypic resistance against the test carbapenems were further screened for the relevant carbapenem-resistance genes using PCR technique and PCR products were viewed using AGE. The list of primers that were used are listed in Table 2.

## 3. Results

### 3.1. Identification and Characterization of Presumptive Klebsiella Spp. Isolates

**Figure 2:**
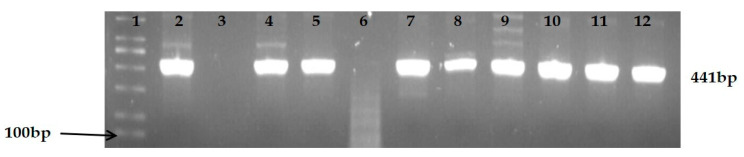
PCR products of the amplification of *gyrA* gene. Lane1: 100 bp molecular weight marker; Lane 2: positive control (ATCC 35657); Lane 3: negative control; Lanes 4–12: positive *Klebsiella* spp. isolates.

**Figure 3:**
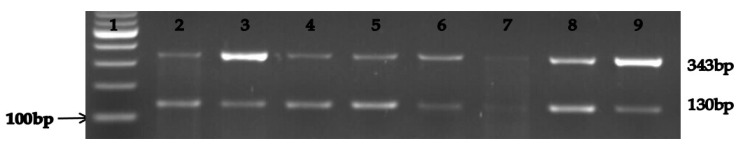
PCR products of the amplification of *pehX* (*K. oxytoca*) and *magA* genes (*Klebsiella pneumoniae*). Lane1: 100 bp molecular weight marker; Lanes 2–9: positive isolates. Band size: 343 bp (*K. oxytoca*); 130 bp (*K. pneumonia*).

A total of 352 presumptive *Klebsiella* spp. isolates were obtained from all the samples analyzed. About 83% (291/352) of the isolates were identified as *Klebsiella* spp. by PCR. The confirmed *Klebsiella* spp. isolates were further characterized by PCR into different species using specific primers. Out of 291 *Klebsiella* spp., a total number of 234 (80%) isolates belonging to the two species identified (*K. pneumonia* 182, 63%; *K. oxytoca* 67, 23%) and unidentified species, 57 (20%). Figure 2 is a representation of the PCR products of the PCR-confirmed *Klebsiella* species while Figure 3 represents the agarose gel electrophoresis of the PCR products of the two *Klebsiella* spp. (*K. pneumonia* and *K. oxytoca*). Table 3 highlights the number of confirmed *Klebsiella* spp. isolated from the different sample types in this study.

### 3.2. Antimicrobial Susceptibility Patterns of the Confirmed Klebsiella Species

**Figure 4:**
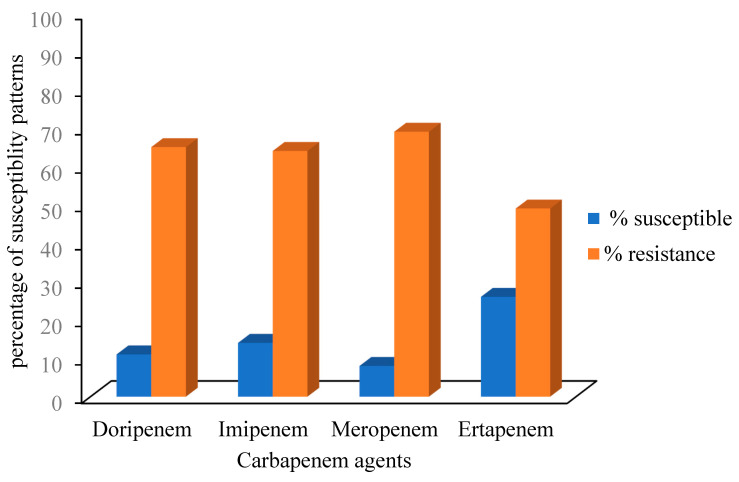
Antimicrobial susceptibility patterns of *Klebsiella* spp. isolates.

All the isolates that were confirmed by PCR techniques were subjected to the carbapenems antibiotics in vitro, meropenem had the highest percentage resistance (161, 69%). The decreasing order of percentage resistance exhibited by the PCR-confirmed *Klebsiella pneumoniae* (182) against the antibiotics tested is as follows; imipenem (93, 51%), ertapenem (64, 35%), doripenem (64, 35%), meropenem (62, 34%) while the percentage resistance for *K. oxytoca* (67) follows the order; meropenem (23, 35%), doripenem (20, 30%), ertapenem (9, 14%), and imipenem (8, 13%). Additionally, all those isolates that showed intermediate were considered as resistant. The antimicrobial susceptibility patterns of the *Klebsiella* spp. isolates recovered from the selected DMs in the Eastern Cape Province of South Africa are shown in Figure 4.

### 3.3. Molecular Characterization of the Relevant Carbapenem Resistance Genes

**Figure 5:**
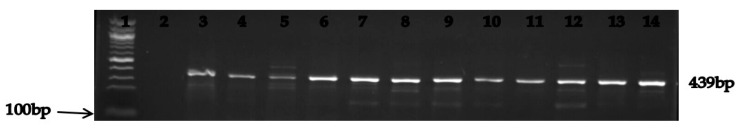
PCR products of the amplification of *bla_NDM-1_* gene. Lane1: 100 bp molecular weight marker; Lane 2: negative control; Lanes 3–14: positive isolates.

**Figure 6:**
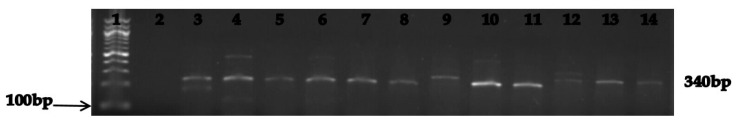
PCR products of the amplification of *bla_KPC_* gene. Lane1: 100 bp molecular weight marker; Lane 2: negative control; Lanes 3–14: positive isolates.

Out of 173 *Klebsiella* spp. that showed resistance against the test antibiotics, 100 (58%) isolates were confirmed by PCR to harbour carbapenem-resistance genes and the result is shown in Table 4 while Table 5 represents the distribution of multiple carbapenem-resistance genes harboured by the isolates. The PCR products of the amplification of *bla_NDM-1_* and *bla_KPC_* genes are shown in Figure 5 and Figure 6, respectively.

### 3.4. Percentage Occurrence of the Antimicrobial Resistance Genes in Klebsiella Species

**Figure 7:**
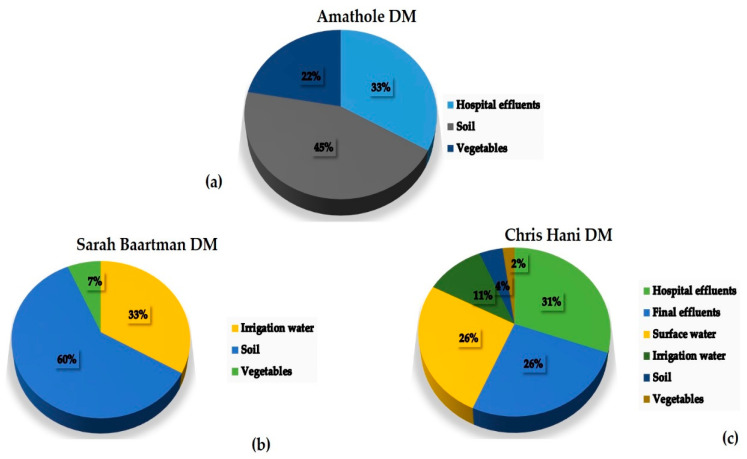
The proportions of occurrence of *Klebsiella* spp. isolates with ARGs recovered from three DMs in the Eastern Cape Province, South Africa. The following is the order of the percentage occurrence of carbapenem-resistance genes in the different sample types; (**a**) hospital effluents (33%), soil (45%) and vegetables (22%); (**b**) irrigation water (33%), soil (60%) and vegetables (7%); (**c**) hospital effluents (31%), final effluents (26%), surface water (26%), irrigation water (11%), soil (4%) and vegetables (2%).

All the *Klebsiella* spp. isolates that were subjected to the carbapenems used were further screened for the presence of carbapenem-resistance genes, soil samples (26; 23%) from the various farms had the highest percentage occurrence of CRGs. Figure 7 shows the proportions of the confirmed *Klebsiella* species harbouring ARGs in the different environmental samples collected. Out of 23 hospital effluent isolates that harboured *bla_NDM-1_* gene, 20 were recovered from Chris Hani DM while three of the isolates were recovered from Amathole DM with five of those isolates harbouring both *bla_NDM-1_* and *bla_KPC_* genes. Results also showed all the CRE isolates from WWTP (11) were from Chris Hani DM and eight harboured *bla_NDM-1_* gene, three harbored *bla_KPC_* gene and one isolate harboured both *bla_NDM-1_* and *bla_KPC_* genes. Klebsiella isolates recovered from surface water (19) showed 12 isolates were recovered from Chris Hani with one isolate harbouring the three CRGs, two isolates harbouring *bla_NDM-1_* and *bla_KPC_* genes, while seven isolates were recovered from Amathole. Sarah Baartman DM (14, 36%) had the highest overall percentage of CRE isolates from farm samples (i.e., irrigation water, soil and vegetable samples).

## 4. Discussion

Different environmental samples collected from the selected sampling sites within the Eastern Cape Province (ECP), South Africa were analyzed in order to detect and characterize the recovered Klebsiella isolates and to screen the phenotypically resistant *Klebsiella* spp. for the relevant carbapenem-resistance genes (CRGs). Among the total number (352) of presumptive *Klebsiella* spp. isolates, 291 (83%) were confirmed positive by molecular techniques, a very high percentage occurrence which is in conflict with the report of Rahman et al. [26], where only 14% were confirmed *Klebsiella* spp. from clinical isolates. This can be as a result of the number of isolates recovered from different niches in our study and also considering the moderately high prevalence of *K. oxytoca* among the confirmed species, more so, studies by Jacob et al. [27] and Trivedi et al. [28] also reported the presence of *K. oxytoca* from environmental isolates. In our present study, among the confirmed *Klebsiella* isolates, *Klebsiella pneumonia* (182, 63%) had the higher number of isolates than *K. oxytoca* (67, 23%) and this is in accordance with a study by Siri et al. [29] (2011). Other studies like Sharma et al. [30] and Samanta et al. [31] also reported the occurrence of *Klebsiella* species in isolates from environmental samples.

The number of reservoirs for CRE is increasing, not only in hospitals, but also in the community, among animals (e.g., livestock, companion animals, and wildlife) and in the environment. In this study, Amathole and Sarah Baartman DMs were observed to have elevated percentage occurrence of carbapenem-resistant *Klebsiella* spp. isolates recovered from farm soil samples. Generally, the presence of CRKP obtained from the different sampling sites of the study area can be of serious health concern. In this study, the highest percentage of resistance (69%) was against meropenem which is in contrary to the study by Peneş et al. [32], who reported slightly lower resistance against meropenem and imipenem. Another study by Ben Tanfous et al. [33] reports ertapenem showing high activity against *Klebsiella* spp. and that report is in corroboration with our findings where the Klebsiella isolates showed the lowest resistance against ertapenem (74%). Some other studies [34,35] reported *Klebsiella pneumonia* with high percentage of resistance against doripenem and imipenem and these are also in accordance with our present study with both antibiotics having resistance percentages of 89% and 86%, respectively.

Enterobacteriaceae members such as *Enterobacter* spp., *Escherichia coli*, and *Klebsiella* spp. have been reported to be associated with exceedingly high levels of antimicrobial resistance (AR) and *K. pneumonia* is among the group to have exhibited high resistance against carbapenems [36]. Among the various members of CRE, CRKP have received the most attention because they have the greatest potential to contribute to the overall problem of AR [37,38]. In a study conducted in Egypt, ElMahallawy et al. [39] reported the detection of OXA-48-like positive CRE in clinical isolates, which emphasized the prevalence of CRE in sub-Sahara Africa and this calls for more studies to be carried out in the region. In South Africa, reports on the presence of ARGs (specifically carbapenemase-producing genes) in isolates from different environmental niches are scarce, however, various studies on the high occurrence of ESBL have been previously reported [40,41,42].

Some of the positive isolates were phenotypically susceptible to one or more of the selected antibiotics but appeared to possess ARGs, suggesting the possibility of one silent gene. Our findings are in agreement with the findings of Lamba and Ahammad [43] and another report of Ng et al. [44] revealing high prevalence of clinically relevant ARGs in wastewaters. However, isolates that exhibited phenotypic resistance but lacked ARGs and vice versa can be as a result of antimicrobial resistance mediated by non-described or included genes and the limitations involved in the use of PCR to detect these genes. Interestingly, ESBL-producing Enterobacteriaceae in vegetables, soil, and water of the farm environment was reported in study by Said et al. [45], however there is a dearth of information on the presence of CRE harbouring *bla_NDM_*, *bla_KPC_*_,_ and *bla_OXA-48-like_* genes in isolates recovered from wastewater, hospital sewage, lakes, and farm samples within the ECP, South Africa.

To the best of our knowledge, this present study is the first report investigating environmental bacterial isolates of Enterobacteriaceae family harbouring significant CRGs such as *bla_NDM_*, *bla_KPC_*, and *bla_OXA-48-like_* within major DMs in the ECP, South Africa. Our findings show that soil samples generally had the most strains of *Klebsiella* spp. harbouring ARGs (89%) in comparison with other sample types from all DMs. This may be as a result of the link between hospitals, WWTPs and receiving surface water used for agricultural purposes, a common practice in rural areas [46]. However, among the bacterial isolates that exhibited multiple resistance against carbapenems, our results show a low percentage of the isolates harbouring the *bla_OXA-48-like_* gene (6%) and our findings are in accordance with another report by Mairi et al. [47]. Among the *Klebsiella* spp. isolates screened for the presence of ARGs, only one *K. pneumonia* isolate recovered from river water in Chris Hani DM showed the presence of all three CRGs (*bla_NDM_*, *bla_KPC_*, *bla_OXA-48-like_*) while 15 *Klebsiella* spp. isolates (from 8 hospital effluents, 3 farm soils, 2 rivers, 1 final effluent, 1 vegetable) were positive for genes of two different CRGs (12 *bla_NDM_* and *bla_KPC_*; 3 *bla_NDM_* and *bla_OXA-48-like_*). In our study, 17 isolates that exhibited multiple resistance were recovered from Chris Hani DM while only three isolates from farm soil were recovered from Sarah Baartman DM. This may be as a result of industrial activities in the different geographic areas with Chris Hani DM having the highest number of hospitals and industries. Among the few recent studies carried out on the prevalence of CRE, Hoang et al. [48] reported similar distributions of NDM and KPC-producing *Klebsiella* spp. in hospital and aquatic isolates in Vietnam which supports our present study. Bacterial isolates recovered from surface water and irrigation water had relatively high percentages of ARGs unlike hospital effluent and WWTPs final effluents, which could be as a result of the sample size in this study. Vegetable samples had the lowest percentage of isolates that harboured ARGs in the selected DMs. Our findings showed Enterobacteriaceae group (particularly *Klebsiella* spp.) perhaps already effectively resistant against carbapenems.

In conclusion, this study evidences the risk of prevalence of potential carbapenem resistant *Klebsiella* spp. in the environments of the Eastern Cape Province. The detection of carbapenem-resistance genes in pathogenic isolates of *Klebsiella* spp. recovered from hospital effluents, WWTPs, and river samples indicates that it could be as a result of improper treatment of final effluent before discharge into receiving aquatic environment. Furthermore, the occurrence of ARB in isolates recovered from farm samples may be associated with the possibility of transmission of ARGs due to the use of manure in farming and receiving watershed as a source of irrigation water and this is a concern to public health. Our findings strongly point out the need for surveillance, control, and management of antimicrobial agents use as well as calls for more studies in this area. Also, rapid and reliable detection of CP-CRE is critical for preventing their further spread in these settings.

## Figures and Tables

**Table 1:** Primer sequences of target species and their respective amplicon sizes and PCR cycling conditions.

Target Strain	Target Gene	Primer Sequence (5′→3′)	Amplicon Size (bp)	PCR Cycling Condition	References
Klebsiella genus	*gyrA*	F: CGC GTA CTA TAC GCC ATG AAC GTA R: ACC GTT GAT CAC TTC GGT CAGG	441	Initial denaturation of 5 min at 94 °C followed by 35 cycles, denaturation at 94 °C for 30 s, annealing at 55 °C for 45 s, extension at 72 °C for 45 s and final extension at 72 °C for 10 min.	Salloum et al. [24]
*Klebsiella pneumonia*	*magA*	F: ATT TGA AGA GGT TGC AAA CGAT R: TTC ACT CTG AAG TTT TCT TGT GTTC	130	Initial denaturation of 5 min at 94 °C followed by 35 cycles, denaturation at 94 °C for 30 s, annealing at 55 °C for 30 s, extension at 72 °C for 40 s and final extension at 72 °C for 10 min.	Salloum et al. [24]
*Klebsiella oxytoca*	*pehX*	F: GAT ACG GAG TAT GCC TTT ACG GTG R: TAG CCT TTA TCA AGC GGA TAC TGG	343	Initial denaturation of 5 min at 94 °C followed by 35 cycles, denaturation at 94 °C for 30 s, annealing at 55 °C for 30 s, extension at 72 °C for 40 s and final extension at 72 °C for 10 min.	Salloum et al. [24]

**Table 2:** List of primers for screening of carbapenem resistance genes [24].

Target Gene	Primer Sequence (5′→3′)	Amplicon Size (bp)	PCR Cycling Condition
*bla_NDM-1_*	F: AAA ACG GCA AGA AAA AGC AG R: AAA ACG GCA AGA AAA AGC AG	439	Initial denaturation of 5 min at 95 °C followed by 35 cycles, denaturation at 95 °C for 1 min, annealing at 52 °C for 1 min, extension at 72 °C for 1 min and final extension at 72 °C for 5 min.
*bla_KPC_*	F: AAA ACG GCA AGA AAA AGC AG R: AAA ACG GCA AGA AAA AGC AG	340	Initial denaturation of 5 min at 95 °C followed by 35 cycles, denaturation at 95 °C for 1 min, annealing at 56 °C for 1 min, extension at 72 °C for 1 min and final extension at 72 °C for 5 min.
*bla_OXA-48-like_*	F: TTGG TGGC ATCG ATTA TCGG R: GAGC ACTT CTTT TGTG ATGG C	597	Initial denaturation of 5 min at 95 °C followed by 35 cycles, denaturation at 95 °C for 1 min, annealing at 56 °C for 1 min, extension at 72 °C for 1 min and final extension at 72 °C for 5 min.

**Table 3:** Number of confirmed *Klebsiella* spp. isolated from different sample types.

Sample Types	Number of Confirmed *K. pneumonia* (cfu/mL)			Number of Confirmed *K. oxytoca* (cfu/mL)		
	Amathole DM	Chris Hani DM	Sarah Baartman DM	Amathole DM	Chris Hani DM	Sarah Baartman DM
Hospital effluent	21	22	-	11	3	-
WWTP final effluent	10	25	-	2	2	-
Surface water	6	26	-	19	8	1
Irrigation water	3	5	14	9	1	7
Farm soil	9	19	10	8	11	7
Vegetables	3	8	1	9	10	7

**Table 4:** Distribution of carbapenem-resistance genes identified among carbapenemase-producing Enterobacteriaceae.

Carbapenem-Resistance Genes	Number of Isolates	Percentage Occurrence (%)
*bla_NDM-1_*	73/173	42
*bla_KPC_*	17/173	10
*bla_OXA-48-like_*	10/173	6
**Total**	**100**	**58%**

**Table 5:** Distribution of multiple carbapenem-resistance genes identified among the isolates.

Patterns of Multiple Resistance Genes	Number of Isolates				Total
	*K. pneumoniae* (%)	*K. oxytoca* (%)	Unidentified Species (%)	Sample Types	
*bla_NDM-1_, bla_KPC_, bla_OXA-48-like_*	0	0	1	River water	1
*bla_NDM-1_, bla_KPC_*	2	2	8	Hospital effluent, farm soil, WWTP, river water	12
*bla_KPC_, bla_OXA-48-like_*	1	1	4	Farm soil	6
*bla_NDM-1_, bla_OXA-48-like_*	0	1	0	Vegetable	1
